# Colorectal cancer susceptibility: apparent gender-related modulation by *ABCB1* gene polymorphisms

**DOI:** 10.1186/s12929-014-0089-8

**Published:** 2014-09-04

**Authors:** Marcella Martinelli, Luca Scapoli, Francesca Cura, Maria Teresa Rodia, Giampaolo Ugolini, Isacco Montroni, Rossella Solmi

**Affiliations:** Department of Experimental, Diagnostic and Specialty Medicine, University of Bologna, Via Belmeloro, 8-40126 Bologna, Italy; Centre of Molecular Genetics, “CARISBO Foundation”, University of Bologna, Bologna, Italy; Department of Medical and Surgical Sciences, University of Bologna, Bologna, Italy

**Keywords:** Colorectal cancer, *ABCB1* gene, Polymorphism, Association analysis

## Abstract

**Background:**

The ATP-binding cassette transporter B1 (*ABCB1*) gene codes for a membrane efflux pump localized in epithelial cells. Together with other Permeability-glycoproteins in the small and large intestine, its product represents a barrier against xenobiotics, bacterial toxins, drugs and other substances introduced with diet, including carcinogens. The aim of this investigation was to verify the possible contribution of *ABCB1* single nucleotide polymorphisms (SNPs) to the genetic risk of colorectal cancer (CRC).

**Results:**

DNA obtained from the peripheral blood of 98 CRC patients and 100 healthy controls was genotyped for the three selected SNPs: 1236C > T (rs1128503), 2677G > T/A (rs2032582), and 3435C > T (rs1045642). Molecular data were analyzed to asses allele and haplotype association with CRC.

No evidence of an association between *ABCB1* alleles and CRC occurrence as a whole was found. However, *ABCB1* showed either association with carcinoma of the sigmoid colon, and appeared able to influence the sex ratio among CRC patients. These two effects seemed to act independently based on multivariate analysis. We showed that *ABCB1* polymorphisms were able to influence CRC susceptibility related to tumor localization and patient gender.

**Conclusions:**

We suggest that sensitivity to undetermined risk factors could depend on the genetic background of *ABCB1* locus, with a mechanism that also depends on patient gender.

**Electronic supplementary material:**

The online version of this article (doi:10.1186/s12929-014-0089-8) contains supplementary material, which is available to authorized users.

## Background

Colorectal cancer (CRC) is among the three leading causes of mortality determined by malignancies in humans worldwide and the second cause of death in Europe [1,2]. Genetic susceptibility factors seem to interact with environmental – in particular, diet-related and smoking-related – factors to increase the risk of CRC [3,4]. Over 50% of CRC etiology has been demonstrated to be attributable to diet and lifestyle [5,6]. A number of studies has recently been carried out to verify the interaction between specific nutrients (meat, fish, fruit and vegetables, fibre, vitamins, alcohol) and susceptibility genes [7]. The intestinal epithelium is the key point of interaction between substances introduced through the diet and gene products involved in the human metabolism. The adenosine triphosphate-binding cassette transporter sub-family B member 1, *ABCB1* (also called multiple drug resistance 1 - *MDR1*) plays a role in modulating this interaction. Expressed in the gastrointestinal tract in particular, it creates a sort of barrier aimed at regulating the uptake of metabolites and drugs [8]. Toxic xenobiotics specifically are eliminated by organs which have an excretory function like the liver and kidneys.

The Permeability-glycoprotein (P-gp), coded by *ABCB1* gene, belongs to a group of ATP-dependent efflux pumps that selectively transport substances out of cells. The transport of harmful substances is necessary to protect cells from death [9]; however, in a mouse model, mdr1a overexpression has been associated with apoptosis inhibition and increased risk of cancer [10]. More generally, an altered function of P-gp could create a perturbation of the intra-extra cellular environment equilibrium that could lead to an increased risk of internalization of DNA damaging factors, or potential carcinogens, thereby modulating susceptibility to neoplasm transformation.

Common *ABCB1* gene polymorphisms have been investigated by different authors because of their potential ability to modulate *ABCB1*-mediated transport. In particular, the C3435T (rs1045642) variant, that was correlated with altered *ABCB1* expression and activity in an *in vivo* functional study [11], was also found to be associated with an increased risk of developing CRC in patients under the age of 50 [12] and in older non-smokers [13]. In addition, C3435T (rs1045642) and G-rs3789243-A variants have been seen to be associated with a modulation of the risk of CRC, in conjunction with consumption of red and processed meat [14]. However, conflicting results have been reported, probably due to the intragenic heterogeneity among investigated populations [15–17], and no association with cancer susceptibility was found in a meta-analysis considering 34 case–control studies, of which 9 were CRC-control studies [17].

In order to evaluate the possible correlation between *ABCB1* polymorphisms and the risk of CRC in a sample study of Italians, we performed a case control association analysis with three SNPs mapping in this gene.

## Methods

### Sample study

A cohort of 98 unrelated Italian colorectal cancer patients was recruited by the Department of Medical and Surgical Sciences of S. Orsola-Malpighi Polyclinic, Bologna University. Diagnosis of colorectal cancer was confirmed by histopathologic examination. The control group consisted of 100 unrelated healthy Italian volunteers matching for gender and age with patient group. Patient information is summarized in Table 1. DNA extraction from peripheral whole blood, collected before primary surgery, was performed using a GenElute™ Blood Genomic DNA Kit (Sigma, Milan, Italy). The study was approved by the ethical committee of Sant’Orsola-Malpighi General Hospital and complied with the Helsinki Declaration’s Ethical Principles for Medical Research Involving Human Subjects. Written informed consent was obtained from all patients and healthy control subjects before study entry.

**Table 1:** **Patient and control group information**

		**Cases**	**Controls**	***P*** **value**
Age		70 ± 13	68 ± 12	0.26
Gender	Male	52	49	0.57
	Female	46	51	
Grading	G1	5	-	
	G2	65	-	
	G3	21	-	
	N.D.^a^	7	-	
Position	Cecum	1	-	
	Ascending colon	33	-	
	Transverse colon	9	-	
	Descending colon	12	-	
	Sigmoid colon	20	-	
	Rectum	23	-	

^a^Not Determined.

### Single nucleotide polymorphism genotyping

Three SNPs mapping respectively on exon 12 (1236C > T; rs1128503), 21 (2677G > T/A; rs2032582), and 26 (3435C > T; rs1045642) of the *ABCB1* gene were chosen, based on literature data. The first and last polymorphisms consist in synonymous variants, while the rs2032582 represents a missense variant (Ala893Ser/Thr). Each polymorphism was amplified by PCR using flanking primers, and the products incubated with a restriction endonuclease, *BsuR*I, *Gsa*I and *Dpn*II (CABRU, Milan, Italy) respectively. Details about polymorphisms are summarized on the Additional file 1. Fragments were separated by 10% native polyacrylamide gel electrophoresis, subsequently stained with ethidium bromide (see Additional file 2). To assess the accuracy of the genotyping outputs, one third of randomly selected samples were blindly tested by a second operator.

### Statistical analysis

Power calculation, given the actual sample size and considering the investigated polymorphisms as susceptibility variations, indicated a power over 80% for genotypic odds ratios higher than 2.5 [18]. The distribution of genotypes in patient and control groups was tested for deviations from the Hardy–Weinberg equilibrium using Pearson’s *χ*^2^ test. Genetic association was firstly investigated using a likelihood ratio approach by Unphased software v3.1.5 in a Windows Vista operative system [19]. Odd ratios were calculated in order to estimate the level of association of the rare allele carriers, i.e. heterozygotes versus non-carriers, as well as homozygotes versus non-carriers.

Logistic regression was then used for multivariate analyses employing SPSS software in order to include available clinical data. Regression modeling was adopted because it did not require specific definition of the exposure and confounder variables, since all explanatory variables were treated in the same way.

Linkage disequilibrium between SNPs was calculated using the Haploview program. Since linkage disequilibrium was evident, haplotype association analysis was adopted to further investigate any evidence of genetic association with single SNP obtained in the investigation. The haplotype association analysis was performed with the aid of Unphased software. A specific test for each haplotype was carried out. This test is a score test for a difference in risk between one haplotype and all the others pooled together. This is in contrast with the odds ratios, which were calculated in relation to a single reference haplotype. A permutation test was performed with 10,000 sample replicates, to allow for multiple testing corrections over all the tests done. In each replicate the trait values were randomly shuffled between all subjects.

## Results

### Bivariate analysis

Only 3 genotypes were missing in the genotyping step (call rate > 99%). Genotype frequencies among both cases and controls satisfied the Hardy-Weinberg equilibrium. An association study was performed to test if genetic variants in *ABCB1* were associated with CRC as whole or with any of the subgroups identified according to histological grade, tumor localization (cecum, ascending, transverse and descending colon cancers were pooled and indicated in the table as colon) and patient gender (Tables 2, 3, and 4). Variant alleles appeared not to be associated with the occurrence of CRC nor with histological grade; however, a significant association was observed with carcinoma of the sigmoid colon, and with CRC in the male strata. Indeed the variant alleles of the rs1128503 and rs2032582 were less frequent in patients with a tumor localized in the sigmoid colon (*P* value 0.006 and 0.023, respectively). The calculated odds ratio (OR) for rs1128503 variant allele heterozygote carriers versus non-carriers (OR_het_) was 0.26 (95% C.I. 0.09–0.80), while for homozygote carriers versus non-carriers (OR_hom_) it was 0.21 (95% C.I. 0.04–1.03). The level of association with rs2032582 appears weaker; the calculated ORs were OR_het_ = 0.49 (95% C.I. 0.18–1.32) and OR_hom_ = 0.22 (95% C.I. 0.05–1.09). Stratification by gender showed that rs1128503 was associated to CRC occurrence among males (*P* = 0.03; OR_het_ = 0.49 (95% C.I. 0.20–1.18), OR_hom_ = 0.31 (95% C.I. 0.09–1.00)). The variant allele, that was less frequent in male patients (MAF = 0.32) with respect to controls (MAF = 0.44), was more frequent in female patients (MAF = 0.53); but the difference with respect to female controls was not significant (*P* = 0.12). No difference in allelic frequency was observed between genders within the control group.

**Table 2:** **Genotyping information about SNP rs1128503 mapping on exon 12**

			**Genotype**				
		**N** ^**a**^	**CC**	**CT**	**TT**	**MAF** ^**b**^	***P*** **value**
Controls		100	33	45	22	0.44	*ref.* ^c^
Cases		96	37	38	21	0.42	0.57
Cases	Grade 1	5	2	1	2	0.5	0.76
	Grade 2	64	24	27	13	0.41	0.58
	Grade 3	20	8	7	5	0.43	0.82
Cases	Colon	51	19	21	11	0.42	0.70
	Sigmoid colon	21	14	5	2	0.21	0.0056
	Rectum	24	4	12	8	0.58	0.085
Controls	Male	49	14	24	11	0.47	*ref.*
Cases	Male	52	25	21	6	0.32	0.027
Controls	Female	51	19	21	11	0.42	*ref.*
Cases	Female	44	12	17	15	0.53	0.12

^a^Number of individuals.

^b^Minor Allele Frequency.

^c^reference.

**Table 3:** **Genotyping information about SNP rs2032582 mapping on exon 21**

			**Genotype**				
		**N** ^**a**^	**GG**	**GT**	**TT**	**MAF** ^**b**^	***P*** **value**
Controls		100	32	44	24	0.46	*ref.* ^c^
Cases		98	32	45	21	0.44	0.75
Cases	Grade 1	5	2	1	2	0.5	1.00
	Grade 2	65	20	31	14	0.45	0.91
	Grade 3	21	8	8	5	0.43	0.71
Cases	Colon	52	15	26	11	0.46	0.98
	Sigmoid colon	22	12	8	2	0.27	0.023
	Rectum	24	5	11	8	0.56	0.20
Controls	Male	49	16	21	12	0.46	*ref.*
Cases	Male	52	20	26	6	0.37	0.18
Controls	Female	51	16	23	12	0.46	*ref.*
Cases	Female	46	12	19	15	0.53	0.32

^a^Number of individuals.

^b^Minor Allele Frequency.

^c^reference.

**Table 4:** **Genotyping information about SNP rs1045642 mapping on exon 26**

			**Genotype**				
		**N** ^**a**^	**CC**	**CT**	**TT**	**MAF** ^**b**^	***P*** **value**
Controls		100	29	42	29	0.5	*ref.* ^c^
Cases		97	28	45	24	0.48	0.68
Cases	Grade 1	5	2	1	2	0.5	1.00
	Grade 2	64	17	29	18	0.51	0.89
	Grade 3	21	8	10	3	0.38	0.16
Cases	Colon	51	14	27	10	0.46	0.52
	Sigmoid colon	22	8	7	7	0.48	0.78
	Rectum	24	6	11	7	0.52	0.79
Controls	Male	49	13	20	16	0.53	*ref.*
Cases	Male	51	17	26	8	0.41	0.092
Controls	Female	51	16	22	13	0.47	*ref.*
Cases	Female	46	11	19	16	0.55	0.24

^a^Number of individuals.

^b^Minor Allele Frequency.

^c^reference.

### Multivariate analyses

Since bivariate statistics suggested that *ABCB1* polymorphisms could influence localization of cancer and could act differently in the two genders, a multivariate statistics approach was adopted to understand how *ABCB1* genotype was able to influence CRC localization and sex ratio among CRC patients, considering additional potentially confounding factors. First of all, logistic regression was used to see if the outcome (0 = healthy controls, 1 = CRCs in a specific position, i.e. colon, or sigma, or rectum) could be predicted according to rs1128503 genotype and gender (Table 5). This analysis indicated that carcinoma of the sigmoid colon is associated with rs1128503 and that the gender did not act as a confounding factor. Indeed, the ORs for variant allele carriers compared to non-carriers, corrected for gender, were almost unchanged in relation with crude ORs obtained in the bivariate analysis.

**Table 5:** **Logistic regression analysis to test if sigmoid colon cancer (0 = healthy controls, 1 = sigmoid colon) can be modeled by rs1128503 genotype and gender**

**Independent variable**	**B** ^**a**^	**s.e.** ^**b**^	**Wald** ^**c**^	**d.f.** ^**d**^	***P*** **value**	**OR (95% C.I.)**
rs1128503			7.57	2	0.02	
CT vs CC	-1.35	0.57	5.58	1	0.02	0.26 (0.08–0.79)
TT vs CC	-1.53	0.81	3.60	1	0.06	0.22 (0.04–1.05)
Gender (0 = male, 1 = female)	-0.33	0.50	0.44	1	0.51	0.72 (0.27–1.92)
Constant	-0.70	0.39	3.17	1	0.07	
Model	*χ*2 = 8.59 d.f. = 3 *P* = 0.035					

^a^regression coefficient.

^b^standard error.

^c^Wald test.

^d^degrees of freedom.

The second multivariate analysis was aimed at testing if sex ratio among patients (0 = CRC male patients, 1 = CRC female patients) could be predicted according to tumour position (colon, sigma, rectum), histological grade (G1, G2, G3), age of diagnosis (years), and rs1128503 genotype (Table 6). Among these independent variables, the factor which influenced the sex ratio most strongly was the rs1128503 genotype (*P* = 0.009). The ORs of female CRC patients with rs1128503 variant allele, versus non-carriers, corrected for all the other factors were OR_het_ = 0.11 (95% C.I. 0.03–0.44), OR_hom_ = 0.24 (95% C.I. 0.06–0.87). In addition, a borderline effect was observed for histological grade (*P* = 0.07), where the OR of females with a G3 tumor, versus G1 tumor, corrected for all the other factors was 0.26 (95% C.I. 0.08–0.87).

**Table 6:** **Logistic regression analysis to test if sex ratio among patients (0 = male patients, 1 = female patients) can be modeled by rs1128503 genotype, histological grade, tumor localization, and age of diagnosis**

**Independent variable**	**B** ^**a**^	**s.e.** ^**b**^	**Wald** ^**c**^	**d.f.** ^**d**^	***P*** **value**	**OR (95% C.I.)**
rs1128503			9.52	2.00	0.01	
CT vs CC	-2.22	0.72	9.51	1.00	0.00	0.11 (0.03–0.45)
TT vs CC	-1.45	0.67	4.67	1.00	0.03	0.24 (0.06–0.87)
Grade			5.27	2.00	0.07	
G2 vs G1	-1.91	1.23	2.42	1.00	0.12	0.15 (0.01–1.65)
G3 vs G1	-1.33	0.61	4.75	1.00	0.03	0.26 (0.08–0.87)
Localization			1.18	2.00	0.55	
sigma vs colon	0.61	0.61	1.02	1.00	0.31	1.85 (0.56–6.08)
rectum vs colon	0.68	0.75	0.83	1.00	0.36	1.98 (0.46–8.57)
Age	-0.01	0.02	0.23	1.00	0.63	
Constant	2.54	1.63	2.43	1.00	0.12	
Model	*χ*2 = 16.86 d.f. = 7 P = 0.018					

^a^regression coefficient.

^b^standard error.

^c^Wald test.

^d^degrees of freedom.

### Haplotype association analysis

To further investigate genetic association between *ABCB1* and cancer of the sigmoid colon, a haplotype association analysis was performed (Table 7). Some haplotypes showed higher association than alleles, suggesting that the actual susceptibility polymorphism was not typed. The higher association level was observed for the haplotype rs1128503C- rs2032582G- rs1045642T (*P* value = 0.0007). This haplotype is more frequent among sigmoid colon cancer patients; the odds ratio with respect to the most frequent haplotype was 3.48 (95% C.I. 1.23–9.82). Since several tests were performed in this analysis, a permutation test was conducted to adjust significance levels: the corrected *P* value for rs1128503C- rs2032582G- rs1045642T association was 0.032.

**Table 7:** **Allele and haplotype association analysis for sigmoid colon cancer**

**M1** ^**a**^	**M2** ^**b**^	**M3** ^**c**^	**Case**	**Control**	**Case-Freq**	**Control-Freq**	**OR (95% C.I.)**	**Chisq**	***P*** **value**
C	-	-	33.0	111.0	0.79	0.56	*ref.* ^d^		
T	-	-	9.0	89.0	0.21	0.45	0.34 (0.15–0.75)	7.668	0.0056
-	G	-	32.0	108.0	0.73	0.54	*ref.*		
-	T	-	12.0	92.0	0.27	0.46	0.44 (0.21–0.90)	5.172	0.023
-	-	C	23.0	100.0	0.52	0.50	*ref.*		
-	-	T	21.0	100.0	0.48	0.50	0.91 (0.48–0.76)	0.075	0.78
C	G	-	30.0	97.4	0.71	0.49	*ref.*	7.104	0.0077
C	T	-	3.0	13.6	0.07	0.07	0.72 (0.19–2.68)	0.018	0.89
T	G	-	0.0	10.6	0.00	0.05	not calculable	2.237	0.13
T	T	-	9.0	78.4	0.21	0.39	0.37 (0.16–0.83)	4.916	0.027
C	-	C	22.0	91.0	0.52	0.48	*ref.*	0.277	0.60
C	-	T	11.0	16.0	0.26	0.08	2.84 (1.16–6.98)	10.560	0.0012
T	-	T	9.0	83.0	0.21	0.44	0.45 (0.20–1.03)	7.120	0.0076
-	G	C	21.9	90.3	0.50	0.45	*ref.*	0.328	0.57
-	G	T	10.1	17.7	0.23	0.09	2.37 (0.94–5.92)	7.129	0.0076
-	T	C	1.1	9.7	0.03	0.05	0.49 (0.06–4.09)	0.539	0.46
-	T	T	10.9	82.3	0.25	0.41	0.55 (0.25–1.20)	4.085	0.043
C	G	C	21.0	83.2	0.50	0.44	*ref.*	0.540	0.46
C	G	T	9.0	10.3	0.21	0.05	3.48 (1.23–9.82)	11.390	0.00074
C	T	C	1.0	7.8	0.02	0.04	0.51 (0.06–4.37)	0.289	0.59
C	T	T	2.0	5.7	0.05	0.03	1.38 (0.25–7.65)	0.488	0.49
T	G	T	0.0	7.6	0.00	0.04	not calculable	1.596	0.21
T	T	T	9.0	75.5	0.21	0.40	0.47 (0.20–1.10)	5.173	0.023

^a^M1 1236 C > T rs1128503.

^b^M2 2677 G > T/A rs2032582.

^c^M3 3435 C > T rs1045642.

^d^reference.

Gender specific haplotype occurrence was evaluated among CRC patients. A significant difference between male CRC and female CRC was evident for each of the tested alleles as well as for some of the haplotypes; nominal *P* values and ORs are shown in Table 8. After multiple comparison correction, males confirmed a significantly higher frequency of the common allele of the rs1128503 polymorphism (corrected *P* value = 0.035), as well as of the haplotype rs1128503C-rs2032582G (corrected *P* value = 0.047).

**Table 8:** **CRC male (case) versus CRC female (control) allele and haplotype association analysis**

**M1** ^**a**^	**M2** ^**b**^	**M3** ^**c**^	**Male**	**Female**	**Male-Freq**	**Fem-Freq**	**OR (95% C.I.)**	**Chisq**	***P*** **value**
C	-	-	71	41	0.68	0.47	*ref.* ^d^	-	-
T	-	-	33	47	0.32	0.53	0.40 (0.23–0.73)	9.22	0.0024
-	G	-	66	43	0.63	0.47	*ref.*	-	-
-	T	-	38	49	0.37	0.53	0.50 (0.29–0.90)	5.53	0.019
-	-	C	60	41	0.59	0.45	*ref.*	-	-
-	-	T	42	51	0.41	0.55	0.56 (0.32–0.99)	3.94	0.047
C	G	-	63.8	34.7	0.61	0.39	*ref.*	9.09	0.003
C	T	-	7.2	6.3	0.07	0.07	0.61 (0.19–1.99)	0.00	0.99
T	G	-	2.2	5.3	0.02	0.06	0.22 (0.04–1.21)	1.88	0.17
T	T	-	30.8	41.7	0.30	0.47	0.4 (0.22–0.75)	6.61	0.010
C	-	C	52.5	27.9	0.52	0.32	*ref.*	7.68	0.0056
C	-	T	17.5	13.2	0.17	0.15	0.70 (0.28–1.75)	0.26	0.61
T	-	C	7.5	11.2	0.07	0.13	0.35 (0.11–1.13)	1.49	0.22
T	-	T	24.5	35.9	0.24	0.41	0.36 (0.18–0.72)	6.78	0.009
-	G	C	51.8	33.4	0.51	0.36	*ref.*	4.41	0.036
-	G	T	13.2	9.6	0.13	0.10	0.89 (0.33–2.39)	0.26	0.61
-	T	C	8.2	7.6	0.08	0.08	0.70 (0.22–2.23)	0.02	0.90
-	T	T	28.8	41.4	0.28	0.45	0.45 (0.24–0.86)	5.90	0.015
C	G	C	49.0	27.4	0.50	0.32	*ref.*	6.35	0.012
C	G	T	12.9	7.4	0.13	0.09	0.97 (0.34–2.81)	0.99	0.32
C	T	T	4.1	6.2	0.04	0.07	0.37 (0.10–1.45)	0.71	0.40
T	G	C	2.1	3.2	0.02	0.04	0.37 (0.06–2.39)	0.33	0.57
T	T	C	5.9	7.4	0.06	0.09	0.44 (0.13–1.58)	0.55	0.46
T	T	T	24.0	34.4	0.24	0.40	0.39 (0.19–0.79)	5.33	0.021

^a^M1 1236 C > T rs1128503.

^b^M2 2677 G > T/A rs2032582.

^c^M3 3435 C > T rs1045642.

^d^reference.

## Discussion

The major function of the *ABCB1* product is to extrude a range of different toxic xenobiotic compounds from cells, thus protecting the organism against potentially harmful exposure. ABCB1, as well as other P-glycoproteins, confers protection by limiting xenobiotic absorption along the gastrointestinal tract, and stimulating excretion of these compounds by the liver and kidneys [20,21]. The activity of this efflux pump may be influenced, in part, by the expression levels of the *ABCB1* gene. At present, at least one polymorphism, the C3435T mapping in exon 26 (rs1045642), has been associated with a reduction of the amount of final product of the gene [11,22]. This polymorphism has been suspected to play a role in the development of different kinds of cancer such as glioma, breast cancer and renal epithelial tumors [17,23,24], in addition, of course, to colon cancer [12,25].

Our investigations indicate that *ABCB1* has a role in CRC, particularly in the onset of carcinoma localized in the sigmoid colon. Indeed, the variant allele at rs1128503 and rs2032582 was less frequent in patients with tumors of the sigmoid colon. Since some haplotypes showed a stronger association than the alleles, we could speculate that the allele which is truly responsible has not yet been typed. Association between *ABCB1* haplotypes and CRC was described in a Polish population suggesting a prognostic role in less advanced CRC, for the same genetic markers investigated in the present work [26,27].

In addition, the present investigation showed that polymorphisms of *ABCB1* were differentially represented in males and females. It should be pointed out that the incidence rate of colon cancer differs significantly according to gender (male: female = 2: 1). Gender may reflect the different influence levels of exposure to risk factors, related to occupational exposures, diet, or lifestyle; but the level of susceptibility to a particular risk factor could also be gender specific. For example, Danish homozygote carriers for the C3435T polymorphism showed an increased risk of developing CRC in relation to their red meat consumption [14]. Hormonal levels can also influence gene expression. It is known, for example, that steroid hormones such as progesterone modulate P-gp expression in some tissues, as evidenced in a murine model [28–30].

In the present investigation we found that SNPs in *ABCB1* can influence susceptibility to CRC, but with gender related specificity. In fact, ancestral alleles were more frequently found in CRC males, while variant alleles were more represented in females, even when haplotype occurrence was considered. Allele frequency in controls was similar in the two genders and at intermediate levels between CRC males and females. SNPs may either alter *ABCB1* expression by influencing regulatory sequences that are the target of steroid hormones, or may alter *ABCB1* ability to process specific substances whose intake could be influenced by gender. This could indicate that SNPs in *ABCB1* are able to influence CRC susceptibility, but with a mechanism that could be gender-related. A similar hypothesis could be endorsed by Nakamura and colleagues’ findings [31], which suggested that serum levels of cortisol and aldosterone can be influenced by the common *ABCB1* rs1045642 polymorphism in women.

## Conclusions

Based on our results we could speculate that, even if hormones like progesterone are not transported by P-gp [29], they could in any case modulate the efficiency of the efflux pump, determining the gender variability observed in our sample study.

In addition, we suggest that some of the differences in colon cancer incidence in male and female patients may be due to gender-dependent differences in *ABCB1* hormonal modulation determining a different role of the same polymorphism. Our data, even if related to different markers howbeit mapping on the same gene, are in accord with those reported by Sainz and colleagues [32]. They too evidenced a different level of CRC risk in patients carrying a specific *ABCB1* SNP variant, depending on gender.

However, given the complexity both of the mechanisms involved in CRC development and of the hormone-mediated effects, a more comprehensive investigation employing bigger sample studies is required to fully address the real role of *ABCB1* polymorphisms in CRC.

## Additional files

Additional file 1:
**Polymorphism characteristics.**
Additional file 2:
**Electrophoretic pattern on 10% acrylamide gel of** ***ABCB1*** **PCR-RFLP genotyping assays: a) 1236C > T rs1128503 (exon 12), the PCR product of 237 bp has two restriction sites. b) 2677 G > T/A rs2032582 (exon 21); c) 3435C > T rs1045642 (exon 26). Numbers on the side represent fragments’ length expressed in base pairs.**

## Abbreviations

ABCB1: ATP-binding cassette transporter B1
SNP: Single nucleotide polymorphisms
CRC: Colorectal cancer
P-gp: Permeability-glycoprotein
OR: Odd ratio
MAF: Minor allele frequency
