# Supplementary figures and images for: Colorectal cancer susceptibility: apparent gender-related modulation by ABCB1 gene polymorphisms
Source: J Biomed Sci. 2014 Sep 4;21(1):89. doi: 10.1186/s12929-014-0089-8 (PMC4428509; doi:10.1186/s12929-014-0089-8)

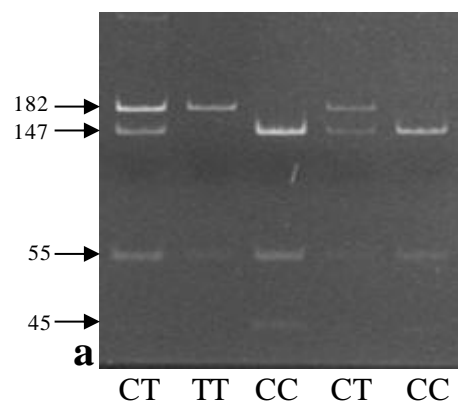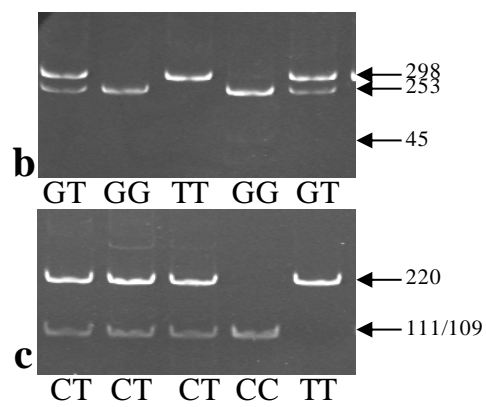

Supplement: Additional file 2: — Electrophoretic pattern on 10% acrylamide gel of ABCB1 PCR-RFLP genotyping assays: a) 1236C > T rs1128503 (exon 12), the PCR product of 237 bp has two restriction sites. b) 2677 G > T/A rs2032582 (exon 21); c) 3435C > T rs1045642 (exon 26). Numbers on the side represent fragments’ length expressed in base pairs. [file 12929_2014_89_MOESM2_ESM.pdf]
